# Inflammatory Myofibroblastic Tumour of the Skull Base

**DOI:** 10.1155/2013/103646

**Published:** 2013-02-26

**Authors:** Jean-Philippe Maire, Sandrine Eimer, François San Galli, Valérie Franco-Vidal, Sigolène Galland-Girodet, Aymeri Huchet, Vincent Darrouzet

**Affiliations:** ^1^Department of Radiation Oncology, Saint-André University Hospital, University Bordeaux Segalen, 33076 Bordeaux, France; ^2^Department of Pathology, Pellegrin University Hospital, University Bordeaux Segalen, Bordeaux, France; ^3^Department of Neurosurgery A, Pellegrin University Hospital, University Bordeaux Segalen, Bordeaux, France; ^4^Department of Otolaryngology and Skull Base Surgery, Pellegrin University Hospital, University Bordeaux Segalen, Bordeaux, France

## Abstract

Inflammatory myofibroblastic tumors (IMTs) are rare benign clinical and pathological entities. IMTs have been described in the lungs, abdomen, retroperitoneum, and extremities but rarely in the head and neck region. A 38-year-old man presented with headache, right exophthalmia, and right 6th nerve palsy. A CT scan revealed enlargement of the right cavernous sinus and osteolytic lesions of the right sphenoid and clivus. MR imaging showed a large tumor of the skull base which was invading the sella turcica, right cavernous sinus, and sphenoidal sinus. A biopsy was performed and revealed an IMT. Corticosteroids were given for 3 months but were inefficient. In the framework of our pluridisciplinary consultation, fractionated conformal radiotherapy (FRT) was indicated at a low dose; 20 Gy in 10 fractions of 2 Gy over 12 days were delivered. Clinical response was complete 3 months after FRT. Radiological response was subtotal 6 months after FRT. Two years later, the patient is well.

## 1. Introduction

Inflammatory myofibroblastic tumors (IMTs), also called inflammatory pseudo tumors or plasma cell granuloma, have been defined in the classification of soft tissue tumors as a lesion composed of myofibroblasts with inflammatory infiltrate [1]. This tumor is now recognized as a neoplastic mass that usually has an uneventful clinical course after radical resection. However, aggressive cases showing invasive, locally recurrent, multiple, and metastatic forms have also been reported [2].

This tumor has been primarily described in the soft tissues and viscera of children and young adults [3], with equal incidence in male and female patients [4]. IMT rarely affects the head and neck region. Despite an apparently benign morphological nature, they have been reported to have locally aggressive growth. Complete surgical excision has proved to be an effective treatment, with some recurrences occurring after surgery. Here, we report a rare case of a large IMT of the skull base causing headache, exophthalmia, and 6th nerve palsy, which could not be operated without heavy morbidity and which resolved after low-dose fractionated radiotherapy.

## 2. Case Report

**Figure 1:**
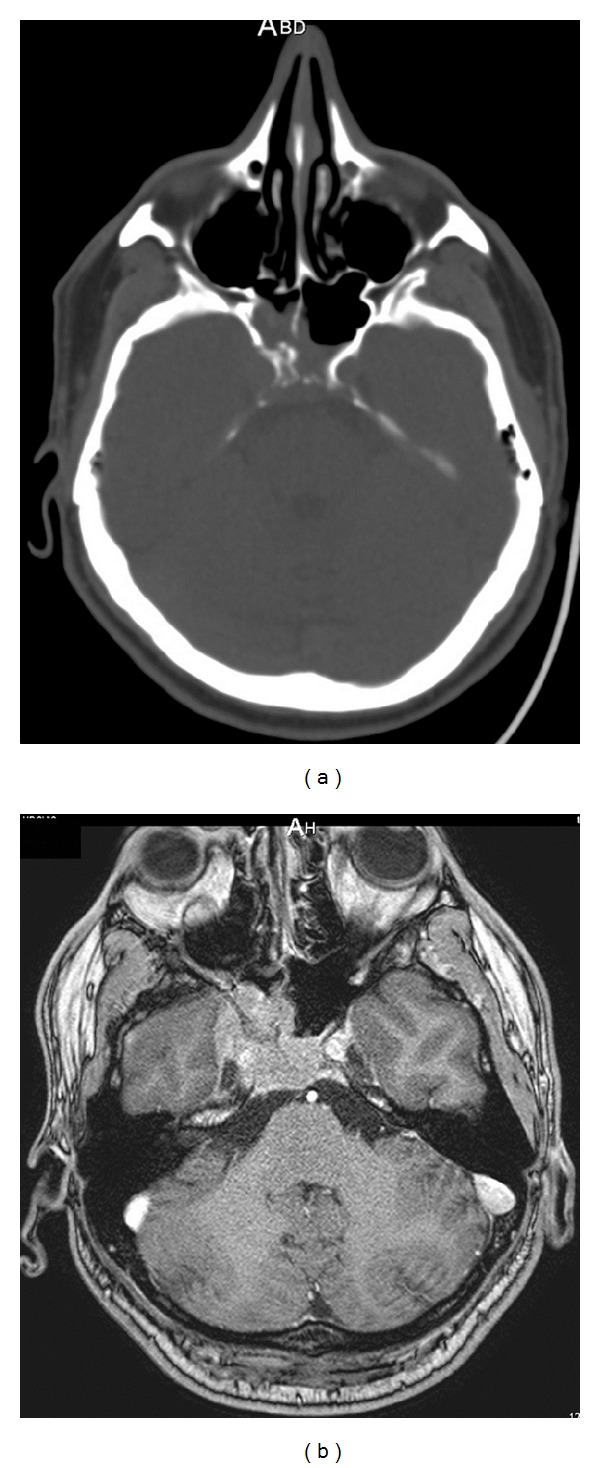
(a) Enlargement of the right cavernous sinus and osteolysis of the right sphenoid bone and clivus on CT scan. (b) Axial T1-weighted MRI with gadolinium contrast showing a large tumor of the skull base invading the sella turcica, right cavernous sinus, and sphenoidal sinus.

**Figure 2:**
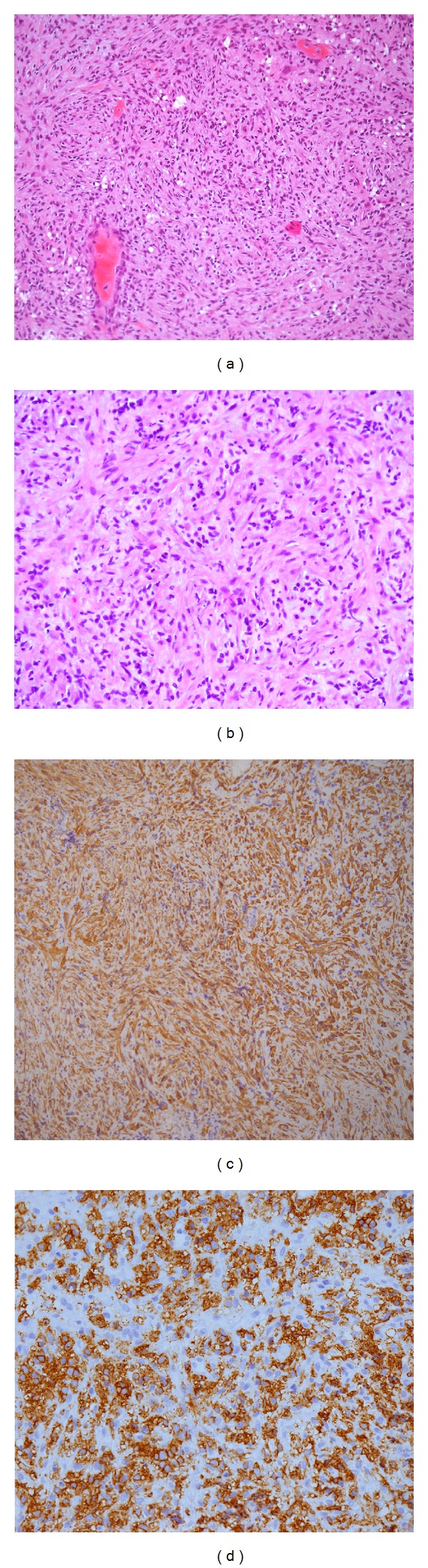
(a) HES × 10: a dense proliferation of spindle cells, partly with an epithelioid aspect, arranged in well-organized fascicles suspended in a myxoid background and admixed with many inflammatory cells, mostly lymphocytes, plasma cells, eosinophils, and rare mast cells. (b) HES × 20: spindle cells present with few atypical features: prominent nucleolus, anisokaryosis, and modest mitotic activity. (c) immunostaining with smooth muscle actin (SMA): spindle cells present a cytoplasmic and diffuse positivity with SMA. (d) immunostaining with CD45 (antipanleukocytes) exhibits the significant lymphoplasmacytic population. FISH analysis was performed: no rearrangement for *ALK-1* gene was noticed.

 A 38-year-old man presented with headache, right exophthalmia, and right 6th nerve palsy. A CT scan revealed enlargement of the right cavernous sinus and osteolytic lesions of right sphenoid and clivus (Figure 1). MR imaging showed a large tumor of the skull base that was invading the sella turcica, right cavernous sinus, and sphenoidal sinus (Figure 1). A biopsy was performed and revealed a dense proliferation of spindle cells, partly with an epithelioid aspect, arranged in well-organized fascicles suspended in a myxoid background, and admixed with many inflammatory cells, mostly lymphocytes, plasma cells, eosinophils and rare mast cells, diagnosed as an IMT (Figure 2). 

 Three months treatment with corticosteroids was inefficient. In the framework of our neurooncological plurisdisciplinary consultation, fractionated conformal radiotherapy (FRT) was indicated at a low dose; 20 Gy in 10 fractions of 2 Gy over 12 days were delivered as corticosteroids were decreased until definitive arrest in 30 days.

**Figure 3:**
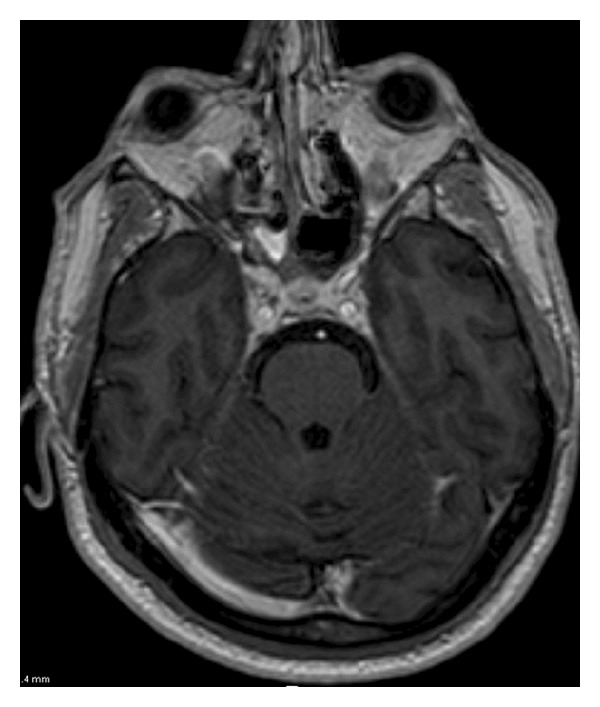
Axial T1-weighted MRI with gadolinium contrast showing a subtotal response 6 months after-radiotherapy.

Clinical response was complete 3 months after FRT. Radiological response was subtotal 6 months after FRT (Figure 3). Two years after FRT, the patient is well and symptom-free. The latest MR imaging confirmed the complete remission.

## 3. Discussion

Our literature review was performed with three key words: inflammatory myofibroblastic tumors (IMTs), inflammatory pseudotumors (IPTs), and Tolosa-Hunt syndrome (THS). IMTs are histologically characterized by dominant myofibroblastic invasion and variable inflammatory infiltration. They are generally benign but sometimes can be locally aggressive. Definition of the 1994 WHO classification of soft-tissue tumors refers to “a tumor composed of differentiated myofibroblastic spindle cells usually accompanied by numerous plasma cells, and/or lymphocytes” [5]. The features of IPTs are also defined by the absence of neoplastic cells or microorganisms and the presence of inflammatory cells with fibrosis. Lymphocytes, plasma cells or eosinophils may predominate in the inflammatory component, or it may be heterogeneous in composition [6]. Tolosa–Hunt syndrome (THS) is defined as an unilateral painful ophthalmoplegia due to chronic benign granulomatous inflammation involving the cavernous sinus and/or the orbital apex and orbit [7–9].

The clinical presentation depends on the location of the tumor. Patients can present with fever, pain, swelling, otorrhea, cranial nerve palsy, and gait disturbance. Radiologically, computed tomography and MRI are both required to assess the extent of bone destruction and infiltration of adjacent structures. Common MRI findings in most reported cases of IMT are low signal intensity on T2-weighted imaging and homogeneous contrast enhancement. They can be multiple [10]. Prompt tissue diagnosis is mandatory to rule out other tumor types such as chordoma, chondrosarcoma, meningioma, metastasis, or giant cell tumor. The site reported in our case is infrequent although some cases have been published [11–19]. Cavernous sinus involvement typically causes painful ophthalmoplegia, that is, TSH [20, 21], but painless ophthalmoplegia can also occur [14].

Low doses of oral corticosteroids are often prescribed and are effective in the management of these tumors [11, 19, 22, 23]. Complete surgical excision of the tumor is also an effective treatment when acceptable, without heavy morbidity [24–27].

Radiotherapy has usually proved to be ineffective [6, 26, 28]. In the series of Lee et al., 8 patients with IPT of the skull base were evaluated; all received initial high-dose corticosteroids, and clinical response was fair. Six patients received low-dose FRT (20 Gy), and most of them did not respond [6]. Their conclusion was that low-dose FRT has a limited role in poor responders to corticosteroid therapy and that higher doses might be a possible alternative strategy.

There have been a few reports regarding high-dose radiation therapy for IPT [29–31]. In the case of Seider et al. the tumor was initially resected, but progression was seen at 1 month of follow-up. Because further surgery to eradicate the tumor completely would have been extensive and disfiguring, 40 Gy FRT were given in 20 fractions [31]. Followup at 27 months showed local control.

Some reports showed 66% to 100% complete remission rates in orbital IPT patients with radiotherapy [32]. Sasagawa et al. reported local control after 20 GY FRT [10]. Other case reports have shown clinical responses after FRT [12, 33–36].

In our case, FRT was indicated since we had previous experience in a case of TSH successfully treated with it [37]. FRT was dramatically efficient after many years of corticosteroid therapy, whereas radical exeresis could not be performed without neurological morbidity for this tumor situated in the cavernous sinus. As in the present case report, clinical and radiological responses were complete. 

The pathogenesis of IMT remains unknown. It has been associated with a number of diseases or agents including Epstein–Barr virus [38] and is thought to result from an exaggerated immunological process [39]. Epstein-Barr viral infections appear to be involved owing to the fact that the virus has been associated with up to 40% of IMT cases [38]. Histopathological examination did not demonstrate any bacteria [11].

## 4. Conclusion

IMT of the skull base is very uncommon and may mimic malignant tumors, so it is important to recognize this entity. The recommended treatment is complete surgical resection with adjuvant corticosteroid treatment. Considering the tumor location, radiotherapy may be a good indication as in our case, even though the efficacy of such treatment is debatable. Further case reports are needed to ascertain the optimal therapeutic regimen.
